# Therapeutic drug monitoring of six contraindicated/co-administered drugs by simple and green RP-HPLC-PDA; application to spiked human plasma

**DOI:** 10.1186/s13065-024-01161-y

**Published:** 2024-04-05

**Authors:** Nada Hesham, Maha A. Hegazy, Hebatallah A. Wagdy

**Affiliations:** 1 Pharmaceutical Chemistry Department, Faculty of Pharmacy, The British University in Egypt (BUE), Cairo, 11837 Egypt; 2 The Health Research Center of Excellence, Drug Research and Development Group, Faculty of Pharmacy, The British University in Egypt, Cairo, 11837 Egypt; 3 Department of Pharmaceutical Analytical Chemistry, Faculty of Pharmacy, Cairo University, Kasr-El Aini Street, Cairo, 11562 Egypt

**Keywords:** Chromatographic analysis, Bioanalytical, Therapeutic drug monitoring, Pharmacovigilance, Imipenem, Meropenem, Ertapenem, Cilastatin, Probenecid, Warfarin, Spiked human plasma, Cmax, Green Chemistry

## Abstract

Therapeutic drug monitoring is an important clinical testing of the drugs to monitor their concentrations in plasma in order to guarantee their optimal impact, and to avoid any side effects resulting from drug-drug interactions. A green reversed-phase high-performance liquid chromatographic method using a photodiode array detector (RP-HPLC-PDA) was developed for the simultaneous determination of three carbapenem antibiotics (Imipenem, ertapenem, and meropenem) with the co-formulated drug (cilastatin) and contraindicated drugs (probenecid and warfarin) in spiked human plasma. The separation was achieved at 25 °C using a gradient elution of a mixture of mobile phase A: methanol and mobile phase B: phosphate buffer (pH 3.0). The photodiode array detector was adjusted at 220 nm. Bioanalytical method validation was carried out as per the FDA guidelines, and the method showed good linearity ranges for the six drugs that included their Cmax levels along with low limits of quantification. Based on the results, the method was found to be accurate and precise; with high % recovery and good % RSD, respectively. The method was successfully applied to spiked human plasma, signifying a good potential to be implemented in future TDM studies of these drugs when co-administered together.

## Introduction

Certain drugs should not be taken concomitantly with each other unless the potential benefits outweigh the risk; that is why proper monitoring of these drugs in plasma is required to ensure optimum therapeutic dosing. Therapeutic drug monitoring (TDM) is a pivotal aspect of pharmacovigilance. It the process of monitoring and quantification of drugs’ serum, or plasma concentrations in order to reduce risks associated with wrong dosing or overdoses and to keep the drugs’ levels within the intended therapeutic window. It is also practiced to prevent the occurrence of adverse side effects and drug-drug interactions (DDIs). Moreover, TDM limits antibiotic resistance in addition to achieving clinical outcomes and controlling patient’s drug dependency. Different analytical techniques are employed in TDM studies. High performance liquid chromatography (HPLC), is the most common and reliable technique in the monitoring and quantification of drugs in biological matrices [1–3].

Carbapenems are a subclass of beta-lactam antibiotics. They are considered to be last-line antibiotics; possessing a broad-spectrum of potent bactericidal effects against gram-negative and gram-positive infections. They are stable against beta-lactamases, and inhibit penicillin-binding proteins. They are mostly administered in severe cases of urinary tract infections, advanced appendicitis, acute pelvic infections, nosocomial and community-acquired pneumonia infections, complicated skin infections, and diabetic foot infections [4]. Additionally, clinical studies reported in literature proved the efficacy of some carbapenems in decreasing the mortality rates in COVID-19 patients with co-infections such as *Acinetobacter baumannii* or *Staphylococcus aureus* strains [5, 6]. The excessive administration of these agents has led to the emergence of resistant carbapenemase-producing species. However, double carbapenem therapies have shown great efficacy against these resistant species as well as multi-resistant gram negative infections [7, 8]. Considering the fact that carbapenems have a very narrow therapeutic index; it is crucial to identify their optimum dosing ranges, with the aim of minimizing adverse drug effects and interactions, and reducing bacterial resistance. For these reasons, carbapenems’ therapeutic drug monitoring is of great importance for achieving dose adjustments in order to reach the desired clinical outcomes, and for minimizing antibiotic resistance [9, 10]. Imipenem (IMP), meropenem (MRP), ertapenem (ETP) are the most common carbapenems in the pharmaceutical market.

**Fig. 1:**
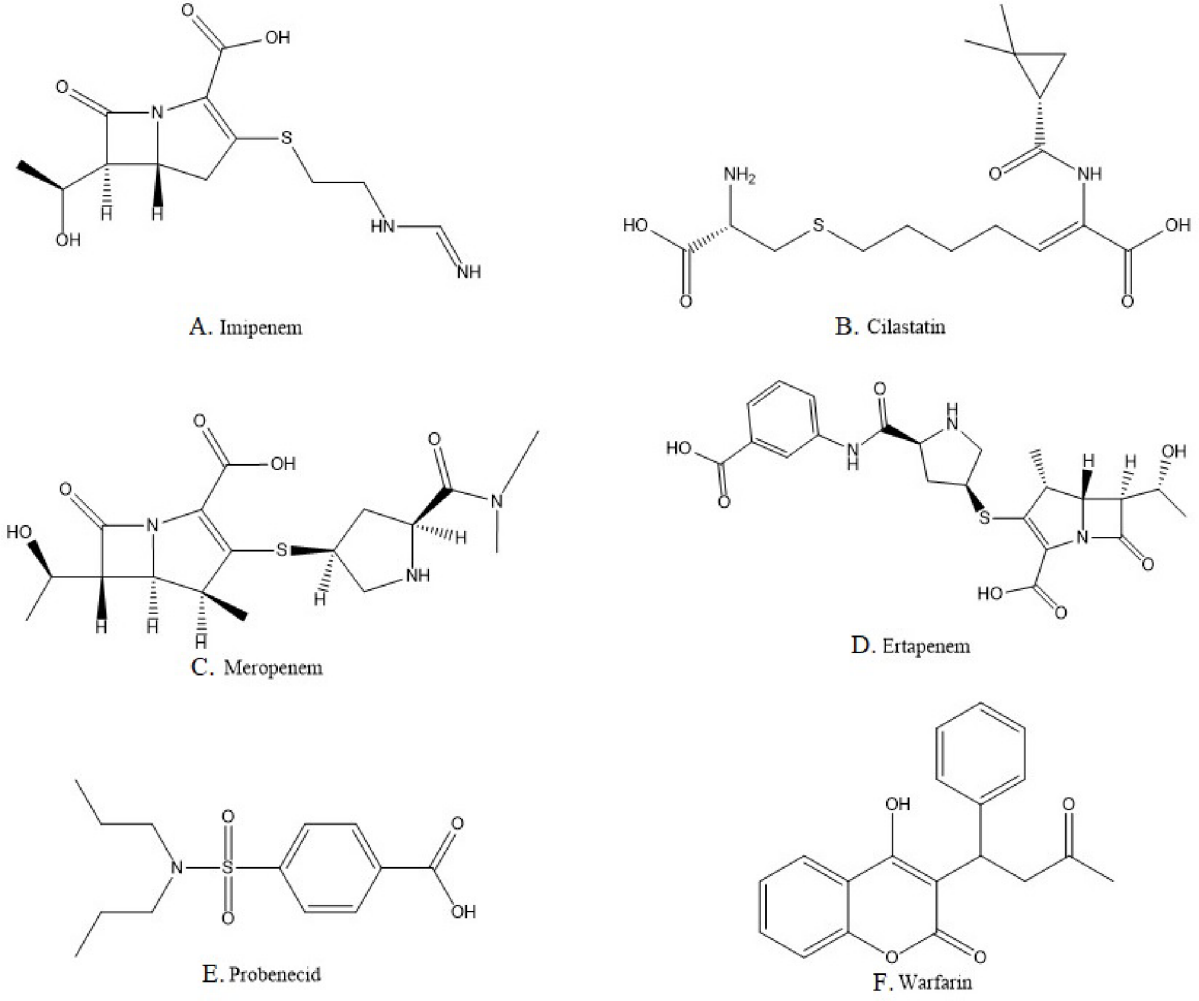
Chemical structures of the drugs under investigation

IMP (Fig. 1.A), (C_12_H_17_N_3_O_4_S) or [(5R,6 S)-3-[2-(aminomethylideneamino)ethylsulfanyl]-6-[(1R)-1-hydroxyethyl]-7-oxo-1-azabicyclo[3.2.0]hept-2-ene-2-carboxylic acid] is the N-formimidoyl derivative of thienamycin; the parent carbapenem. IMP is unstable against renal dehydropeptidase-I (DHP-I) enzyme where it leads to its inactivation, so it is usually administered with DHP-I enzyme inhibitors like cilastatin (CLS) (Fig. 1.B), (C_16_H_26_N_2_O_5_S) or [(Z)-7-[(2R)-2-amino-2-carboxyethyl]sulfanyl-2-[[(1 S)-2,2dimethylcyclopropanecarbonyl]amino]hept-2-enoic acid] [11, 12]. There are many different methods of analysis reported in the literature for the determination of IMP alone, or in the presence of CLS such as electrochemical methods [13], spectrophotometric methods [14], liquid chromatography-mass spectrometry (LC–MS/MS) [15], and high performance liquid chromatography with UV detection (HPLC-UV) [16].

MRP (Fig. 1.C), (C_17_H_25_N_3_O_5_S) or [(4R,5 S,6 S)-3-[(3 S,5 S)-5-(dimethylcarbamoyl)pyrrolidin-3-yl]sulfanyl-6-[(1R)-1-hydroxyethyl]-4-methyl-7-oxo-1-azabicyclo[3.2.0]hept-2-ene-2-carboxylic acid] was later discovered and approved by the Food and Drug Administration (FDA) after IMP. Unlike IMP, MRP is stable against renal DHP-I enzyme, so it does not require to be administered with DHP-I inhibitors [4, 17]. Many methods of analysis are reported in the literature for the determination of MRP, including electrochemical methods [18], spectrophotometric methods [19], spectrofluorimetric methods [20], LC-MS/MS [21], and HPLC–UV [22].

Later, ETP (Fig. 1.D), (C_22_H_25_N_3_O_7_S) or [(4R,5 S,6 S)-3-[(3 S,5 S)-5-[(3-carboxyphenyl)carbamoyl]pyrrolidin-3-yl]sulfanyl-6-[(1R)-1-hydroxyethyl]-4-methyl-7-oxo-1-azabicyclo[3.2.0]hept-2-ene-2-carboxylic acid] was approved by the FDA. It is stable against DHP-I enzyme like MRP, and it has a longer half-life than IMP and MRP, which allows for the once per day administration [4, 23]. According to literature, ETP was determined using different methods of analysis such as electrochemical methods [24], spectrophotometric methods [19],spectrofluorimetric methods [20], LC–MS/MS methods [25], and HPLC–UV [26].

These three carbapenems were found to be contraindicated with probenecid (PRB) according to their FDA labels. PRB (Fig. 1.E), (C_13_H_19_NO_4_S) or [4-(dipropylsulfamoyl)benzoic acid] is a uricosuric agent used usually in combination with other agents like colchicine for the treatment of gout. It increases the excretion of uric acid, and decreases serum urate levels by inhibiting its reabsorption at the proximal tubule [27]. It also has an antiviral and anti-inflammatory effect in viral diseases such as the COVID-19 virus [28]. According to the FDA, PRB increases the plasma level and half-life of IMP when co-administered together [29]. When administered with MRP, it inhibits the latter’s renal excretion as they compete for active tubular secretion, thus increasing plasma levels of MRP, same goes with ETP [30, 31]. There are several methods of analysis reported for the determination of probenecid, including electrochemical methods [32], spectrophotometric methods [33], LC–MS/MS [34], and HPLC–UV [35].

Carbapenems were also found to be contraindicated with warfarin (WRF) as they increase its international normalized ratio, leading to a risk of bleeding. WRF (Fig. 1.F), (C_19_H_16_O_4_), or [4-hydroxy-3-(3-oxo-1-phenylbutyl)chromen-2-one] is an anticoagulant and vitamin K antagonist that is used for treating cases of venous thromboembolism and pulmonary embolism [36]. There are several methods of analysis reported in the literature for the determination of WRF including electrochemical methods [37], spectrophotometric [38], spectrofluorimetric methods [39], and chromatographic methods like: LC-MS/MS [40], and HPLC-UV [41].

The purpose of the present research is to develop and validate a novel reversed phase HPLC method using a photodiode array detector (RP-HPLC-PDA) for simultaneous determination of IMP, ETP, CLS, MRP, PRB and WRF. The method aims for precise, accurate, and environmentally friendly separation with the lowest possible run time for the simultaneous quantification of the studied drugs at their Cmax levels in spiked human plasma, so it can be easily implemented in TDM studies. Additionally, two greenness assessment tools were applied to ensure the greenness of the method; the Green Analytical Procedure Index (GAPI) and the Analytical Eco-Scale (AES). To the best of the authors’ knowledge, no previously published method for the simultaneous determination of these drugs has been reported.

## Materials and methods

### Solvents and reagents

Acetonitrile (ACN) (purity; 99.9%) (Lot: 1,994,099), methanol (MeOH) (purity; 99.8%) (Lot: 2,185,854) and orthophosphoric acid (purity; 85%) (Lot: 1,674,550), (HPLC grade) and potassium phosphate monobasic (purity; ≥99%) (Lot: 1,878,109) (analytical grade) were all supplied from Fisher Chemical, Fisher scientific (Loughborough, Leicestershire, United Kingdom). IMP (purity; ≥98%), CLS (purity; ≥98%), MRP (purity; ≥98%), and ETP (purity; ≥90%) standards were obtained from sigma Aldrich (Darmstadt, Germany). PRB (purity; 99.95%) and WRF (purity; 98%) were thankfully provided by October Pharma, pharmaceutical company (Cairo, Egypt) and GlaxoSmithKline, GSK (Cairo, Egypt), respectively. Plasma was purchased from the Holding Company of Biological Products and Vaccines (Vacsera) (Giza, Egypt).

### Instruments and software

The method was developed using a Thermo Fisher UHPLC Dionex Ultimate 3000 (Germering, Germany) coupled to photodiode array detector model 3000 RS (Germering, Germany) equipped with ISO-3100SD pump, WPS 3000 SL autosampler and a TCC-3000 SD column thermostat. The separation was achieved on a Prontosil Kroma plus^®^ C18 column (150 × 4.6 mm; 5 μm particle size). The software was chromeleon 6.8 (Germering, Germany). Deionized water was obtained from Milli-Q ultrapure water purification system (Thermo Scientific Barnstead Smart2Pure 3 UV, Hungary). Degassing of the mobile phase was carried out using an ultra sonicator (Elmasonic S 60 (H), Germany). The pH of the mobile phase was adjusted using Jenway pH-meter 3310 (Dunmow, Essex, United Kingdom). Samples were centrifuged using (Centurion K241R-United Kingdom). The spiked plasma samples were mixed using a vortex device (Velp scientifica, Europe). Rotatory Vacuum Concentrator (DVP-TYRO 12, Germany) for solvents evaporation equipped with vacuum pump, a solvent trap (CHRIST CT 02–50, Germany) and a rotor (CHRIST RVC 2–18 CDplus, Germany).

### Stock and working standard solutions

Stock standard solutions of 1.0 mg/mL for each of the six drugs were prepared separately depending on each drug’s solubility and stability in various solvents. IMP, MRP, ETP and CLS standard solutions were prepared by dissolving 10.0 mg of each drug in 10 mL deionized water, while PRB and WRF standard solutions were prepared by dissolving 10.0 mg of each drug in 10 mL MeOH to get final concentrations of 1.0 mg/mL. Working standard solutions were prepared by transferring different volumes of each of the six drugs’ standard solutions to a 10-mL volumetric flask, and completing the volume till 10 mL with MeOH to reach a final concentration of 100.0 µg/mL for each drug. The stock standard solutions were freshly prepared.

### Experimental design

Design Expert^®^, trial version 13.0 (Copyright © 2022 Stat-Ease, Inc., Minneapolis, USA) was used for implementing method optimization of the proposed RP-HPLC-PDA method. Central composite design was applied where three numeric factors (% organic phase, pH, flow rate) were set to two levels (low and high) in order to study the impact of these factors on the chromatographic separation as shown in Table 1.

**Table 1:** Factors used in central composite design and their selected levels

Factor	Unit	Low	High	-alpha	+alpha
% of organic phase	%	40.0	60.0	40.0	60.0
pH of the buffer		3.0	7.0	3.0	7.0
Flow rate	mL/min	0.2	0.4	0.2	0.4

### Chromatographic conditions

The chromatographic separation of the drugs under investigation was carried out using C18 column (150 × 4.6 mm; 5 μm particle size). The column oven temperature was set at 25^°^C. The gradient mobile phase consisted of MeOH as solvent A, and 20 mM phosphate buffer (pH 3.0) as solvent B. The buffer was prepared by mixing certain calculated volumes of 20 mM potassium phosphate monobasic and 20 mM orthophosphoric acid, and the pH (3.0) was adjusted by 20 mM orthophosphoric acid. The flow rate ranged from 0.3 to 0.4 mL/min (Table 2). The PDA detection was carried out at 220 nm.

**Table 2:** Optimum gradient elution conditions

Time (min)	Flow rate (mL/min)	%A (MeOH)	%B (Buffer pH 3.0)
0.0	0.4	20.0	80.0
4.0	0.4	45.0	55.0
6.0	0.4	45.0	55.0
7.0	0.4	50.0	50.0
9.0	0.4	50.0	50.0
10.0	0.4	25.0	75.0
12.0	0.4	25.0	75.0
14.0	0.4	90.0	10.0
15.0	0.3	100.0	0.0
30.0	0.3	100.0	0.0
33.0	0.4	20.0	80.0
38.0	0.4	20.0	80.0

### Spiked plasma samples preparation

#### Preparation of calibrator samples

All experimental work in this research was approved by the local ethics committee of the faculty of pharmacy, the British university of Egypt (BUE). Spiked plasma samples were prepared using plasma purchased from the Holding Company of Biological Products and Vaccines (Vacsera) (Giza, Egypt). Calibrators samples were prepared by mixing 100 µL of the corresponding working standard solutions of each drug with 900 µL plasma. 1.5 mL of ACN was transferred to each sample then vortexed for 3 min and centrifuged for 30 min at 6000 rpm. The supernatant was then evaporated and dried using rotatory vacuum concentrator at 60 °C and 1450 rpm for 4 h. At last, the samples were reconstituted with 1 mL MeOH, and transferred to vials to be injected into the HPLC system. The final concentrations are listed in Table 3.

**Table 3:** Calibrators and quality control samples of the six drugs in spiked human plasma

Drugs	Calibrator samples (µg/mL)							QC samples (µg/mL)			
	C1	C2	C3	C4	C5	C6	C7	LLOQ	QCL	QCM	QCH
IMP	10.0	15.0	25.0	35.0	50.0	70.0	100.0	10.0	30.0	40.0	75.0
ETP	15.0	50.0	100.0	150.0	200.0	300.0	400.0	15.0	50.0	140.0	280.0
CLS	10.0	15.0	25.0	35.0	50.0	70.0	100.0	10.0	30.0	40.0	75.0
MRP	5.0	10.0	20.0	30.0	50.0	70.0	100.0	5.0	15.0	35.0	75.0
PRB	5.0	10.0	20.0	30.0	50.0	70.0	100.0	5.0	15.0	35.0	75.0
WRF	1.0	3.0	7.0	10.0	15.0	20.0	30.0	1.0	6.0	12.0	25.0

#### Preparation of quality control samples

Quality control samples used in bio-validation studies included: low (QCL) which is 3 times the lower limit of quantification (LLOQ), QCM (medium) concentration which is 30–50% the highest concentration in the range, and QCH (high) concentration which is 70% of the highest concentration in the range. The QCs were prepared by the same procedure as the calibrators. The final concentrations were (30.0, 40.0, 75.0 µg/mL) for IMP and CLS, (15.0, 35.0, 75.0 µg/ mL) for MRP and PRB, (50.0, 140.0, 280.0 µg/ mL) for ETP and (6.0, 12.0, 25.0 µg/ mL) for WRF, respectively, as listed in Table 3. For the preparation of their laboratory mixture in plasma at their Cmax, certain calculated aliquots of each stock standard solution were transferred to a 10-mL volumetric flask then completed with MeOH. 100 µL of the mixture was mixed with 900 µL plasma, and the sample was prepared as mentioned above so that the final concentration of each drug is corresponding to its Cmax in the mixture as follow: IMP (35.0 µg/ mL), ETP (150.0 µg/ mL), CLS (35.0 µg/ mL), MRP (30.0 µg/ mL), PRB (30.0 µg/ mL), and WRF (10.0 µg/ mL) [42–45].

### Method validation

The method was bio-validated as per FDA guidelines where a number of validation parameters were evaluated including; linearity, selectivity, sensitivity, extraction recovery, accuracy, precision, stability and system suitability [46].

### Linearity and lower limit of quantification (LLOQ)

The linearity of the method was evaluated by constructing a calibration curve for each drug in plasma. A triplicate of seven concentrations were analyzed using the HPLC, and the resulting average peak area from the obtained chromatograms was plotted against the corresponding concentration.

LLOQ is the lowest concentration that can be quantified as per the calibration curve where signal to noise ratio is more than 10 with acceptable accuracy range 80–120%, and % RSD up to 20%.

### Selectivity

Selectivity is the ability of the method to analyze and quantify the drugs without being affected by the interfering substances found in the biological matrix where blank samples should be free of interferences at the analytes’ retention time. It is evaluated using blank samples from six different sources where the response of the interfering substances shouldn’t exceed 20% of the response of the analytes at LLOQ level.

### Accuracy and precision

The triplicate of each quality control (QC) samples was employed to measure both accuracy and precision. Accuracy is defined as the degree of closeness of the results to the true value. Within run and between run accuracy is evaluated using % recovery (%R), where the accepted range is ± 15%, except for LLOQ ± 20%. %R is calculated by Eq. (1) below:

1 $${\text{\% R }}=~\frac{{Found\,concentration}}{{Theoritical\,concentration}} \times 100$$

Precision is the measure of closeness of the results with each other. Within run and between run precision are evaluated by % relative standard deviation (% RSD) where it shouldn’t exceed 15%, and 20% for LLOQ.

### Extraction recovery

Extraction recovery (% Ex. R) is determined to ensure the efficiency and reproducibility of the extraction procedure of the method. It is evaluated by comparing the peak area of pre-extraction QC samples with the post-extraction QC samples where the blank plasma samples are spiked with the drug after plasma extraction at the three QCs concentration levels. It is evaluated by calculating %R using Eq. (2) below:

2 $$\begin{aligned} {\text{\% Ex}}{\text{. R}}= & Average\,peak\,Area\,of\,\operatorname{Pre} \\ & - extraction\,QC\,samples/ \\ & Average\,peak\,Area\,of\,\operatorname{P} ost \\ & - extraction\,QC\,samples \\ & \times 100 \\ \end{aligned}$$

### Stability

Stability is determined by comparing the QCs with freshly prepared ones. Stability studies include benchtop studies, freeze and thaw stability, auto sampler stability, short term stability and long-term stability. The results are assessed by % deviation, where the accepted range is ± 15%. It is calculated by Eq. (3) below:

3 $$\begin{aligned} {\text{\% Deviation}}\,= & \,\% \,recovery\,of\,the\,old\,QC\,samples \\ & -\,\% \,recovery\,of\,the\,fresh\,QC\,samples/ \\ & \,\% \,recovery\,of\,the\,fresh\,QC\,samples \\ & \times {\text{100}} \\ \end{aligned}$$

Benchtop (short term) stability was determined by analyzing QC samples prepared and left in room temperature for 2 h (maximum period for sample preparation), and then compared with fresh QC samples.

Freeze and thaw stability was assessed by subjecting QC samples to three cycles of freezing and thawing. The cycle duration was 24 h, and the samples were stored in a freezer at 4 °C. After analysis, the samples are compared with freshly prepared ones.

In autosampler stability, QC samples were kept in the autosampler for the maximum period of time they can be stored in there (24 h at 25 °C), and then they were analyzed and compared with fresh QC samples.

For assessing long term stability, QC samples were stored (in a freezer at 4 °C) for a long period of time exceeding the time of the whole experiment (28 days). Then they were analyzed and compared with freshly prepared QC samples.

### System suitability

System suitability involves a number of parameters that were investigated to ensure the integrity of the developed method, and the efficiency of the analysis. The studied parameters were: retention time (t_R_), capacity factor (K’), asymmetry factor (A_f_), tailing factor (T_f_), resolution (R_s_), and column efficiency (N).

## Results and discussion

Developing chromatographic methods to be applied in TDM studies is of a great importance in order to assess and monitor drugs’ concentration and possible drug-drug interactions. The chromatographic separation of IMP, ETP, CLS, MRP, PRB and WRF simultaneously in spiked human plasma at their Cmax was challenging; Therefore, method optimization was carried out in order to reach the optimum conditions for separation with high resolution. Moreover, the developed method was bio-validated as per the FDA guidelines. Two greenness assessment tools; the green analytical procedure index (GAPI) and the analytical eco-scale (AES), were applied to ensure the greenness of the method.

### Method optimization

For method optimization, several chromatographic factors were studied to reach the optimum conditions for high resolution separation. Initially, preliminary runs were conducted to evaluate the impact of these parameters on separation. The type of the eluent was assessed, in which two organic solvents were investigated: ACN and MeOH. The latter showed better results and sharper peaks as well as being greener than ACN. 20 mM phosphate buffer (pH 3.0 adjusted using orthophosphoric acid) was selected as the aqueous phase since it possesses low cut off. Low buffer concentration was used to avoid column back pressure and salting out. The effect of temperature was studied at 25 °C, 35 and 45 °C. However, better resolution was obtained at 25 °C. Two columns were studied; Thermo Fisher BDS Hypersil C18 (100 × 3 mm, 3 μm particle size) and Prontosil Kroma plus C18 (150 × 4.6; 5 μm particle size) where the latter showed better resolution and sharper peaks. Different detection wavelengths were examined according to the reported λ_max_ of the six drugs the in literature and their UV spectrums. 220 nm was the selected detection wavelength since it has the highest sensitivity for the six drugs under investigation. The studied parameters served as a starting point for the input runs to be supplied to the design expert^®^, trial version 13.0 (Copyright© 2022 Stat-Ease, Inc., Minneapolis, USA).

**Fig. 2:**
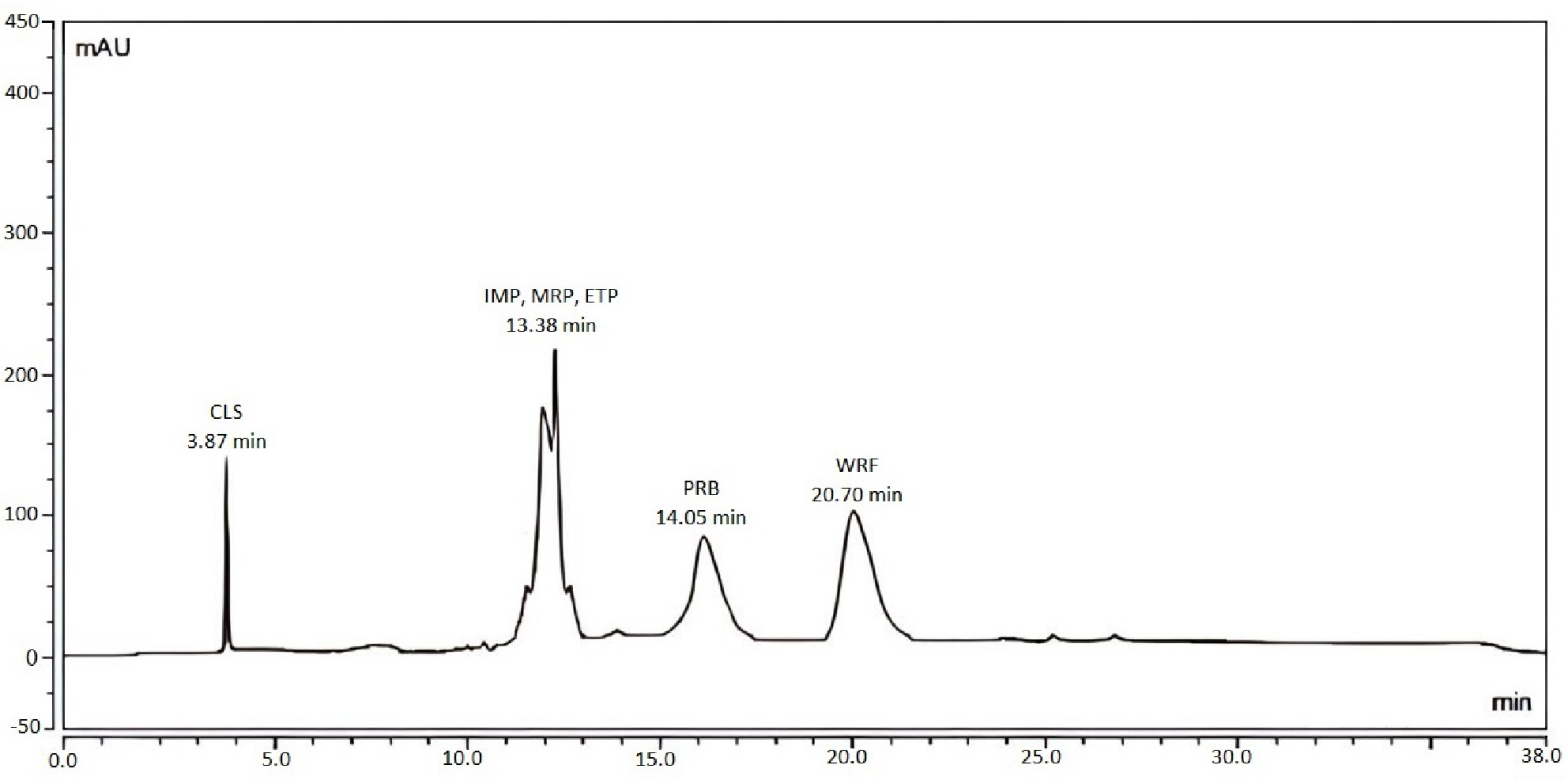
Chromatogram of the six drugs in mixture under the final generated suggested conditions from the Design Expert^®^

The central composite design was adopted to reach the best optimum conditions for the separation of the six drugs using isocratic elution. The three evaluated factors were: the effect of pH, flow rate, and percentage of organic phase. Each factor was studied at two levels (low and high) as shown in Table 1. The software has generated twenty trials and the resulted data from each run was used as an input for the prediction of the optimum conditions (Table 4). The software’s recommended conditions were as follows: the % of the organic phase was 49.23%, the flow rate was 0.4, and the pH was 4.88. Upon applying the suggested conditions, a chromatogram was obtained for the six drugs (Fig. 2), showing the three carbapenems co-eluted together. Even though the predicted method has failed to separate the six drugs efficiently, it gave some insight for method optimization and in the selection of the optimum conditions.

**Table 4:** Design expert generated runs according to selected factors

Run	Factor 1	Factor 2	Factor 3
	A:% organic phase	B:pH Buffer	C:Flow Rate
1	40.0	5.0	0.3
3	50.0	5.0	0.3
4	50.0	5.0	0.2
6	50.0	5.0	0.3
8	50.0	5.0	0.3
10	50.0	5.0	0.3
11	50.0	5.0	0.3
12	50.0	5.0	0.4
15	50.0	5.0	0.3
7	60.0	5.0	0.3
2	40.0	3.0	0.2
5	40.0	3.0	0.4
17	50.0	3.0	0.3
9	60.0	3.0	0.4
14	60.0	3.0	0.2
18	40.0	7.0	0.2
20	40.0	7.0	0.4
16	50.0	7.0	0.3
13	60.0	7.0	0.2
19	60.0	7.0	0.4

**Fig. 3:**
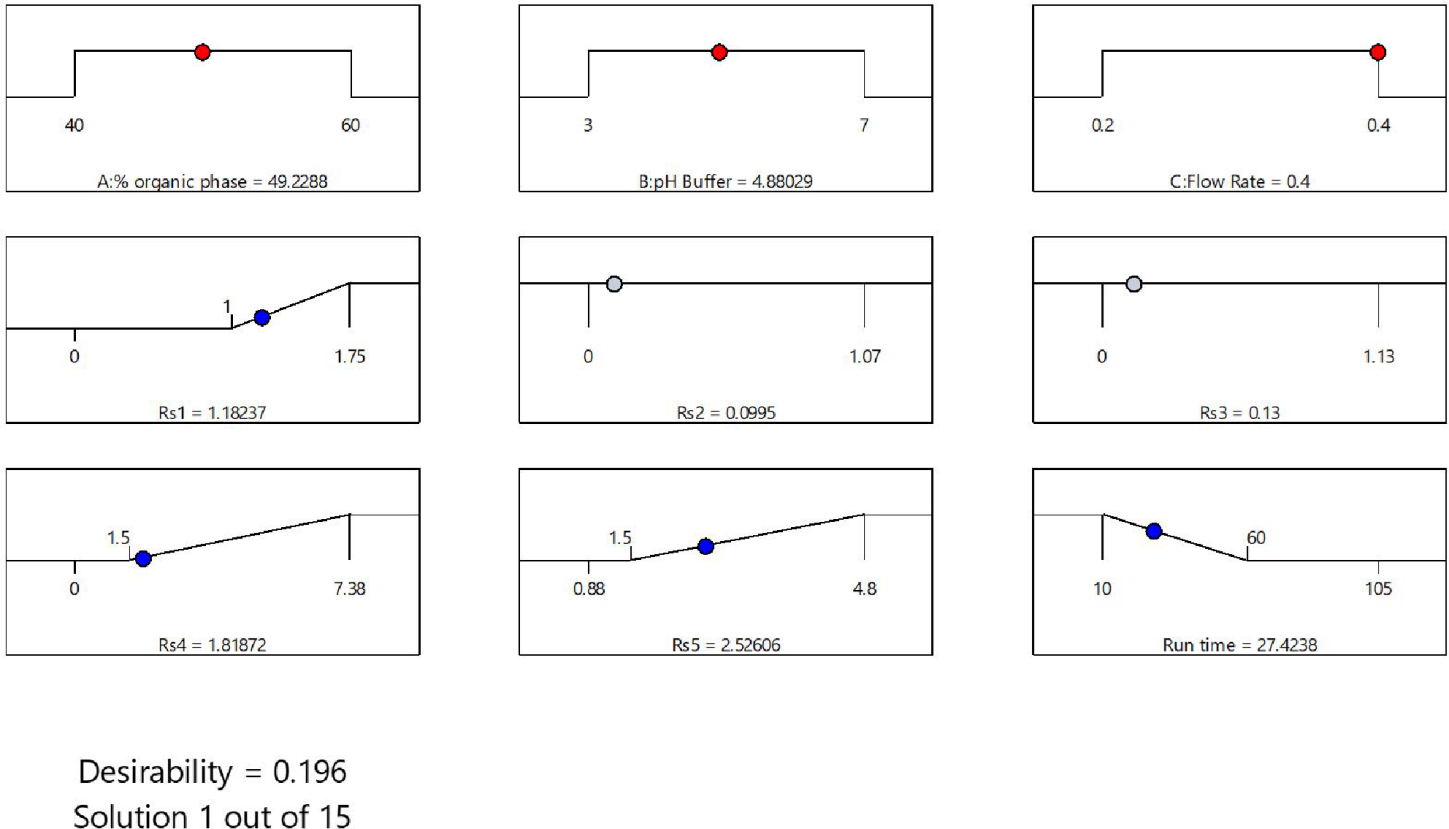
Numerical plot for the suggested conditions by the Design Expert^®^

Since applying the isocratic mode failed to completely separate the six drugs, gradient elution mode was adopted and it was based upon the final data collected from the software’s predicted method (Fig. 3). To achieve the highest resolution, various gradient systems with varying runtimes have been evaluated. Step-wise gradient elution mode was used in which the gradient started with a high ratio of the aqueous buffer phase and decreased as the concentration of the strong mobile phase (MeOH) increased. Moreover, the flow rate of 0.4 mL/min was selected based on the software-suggested data, but the resolution between WRF and PRB was inadequate. In order to resolve this issue, the flow rate was maintained at 0.4 mL/min, and then changed to 0.3 mL/min just before the elution of the two peaks in order to separate them with higher resolution (Table 2).

**Fig. 4:**
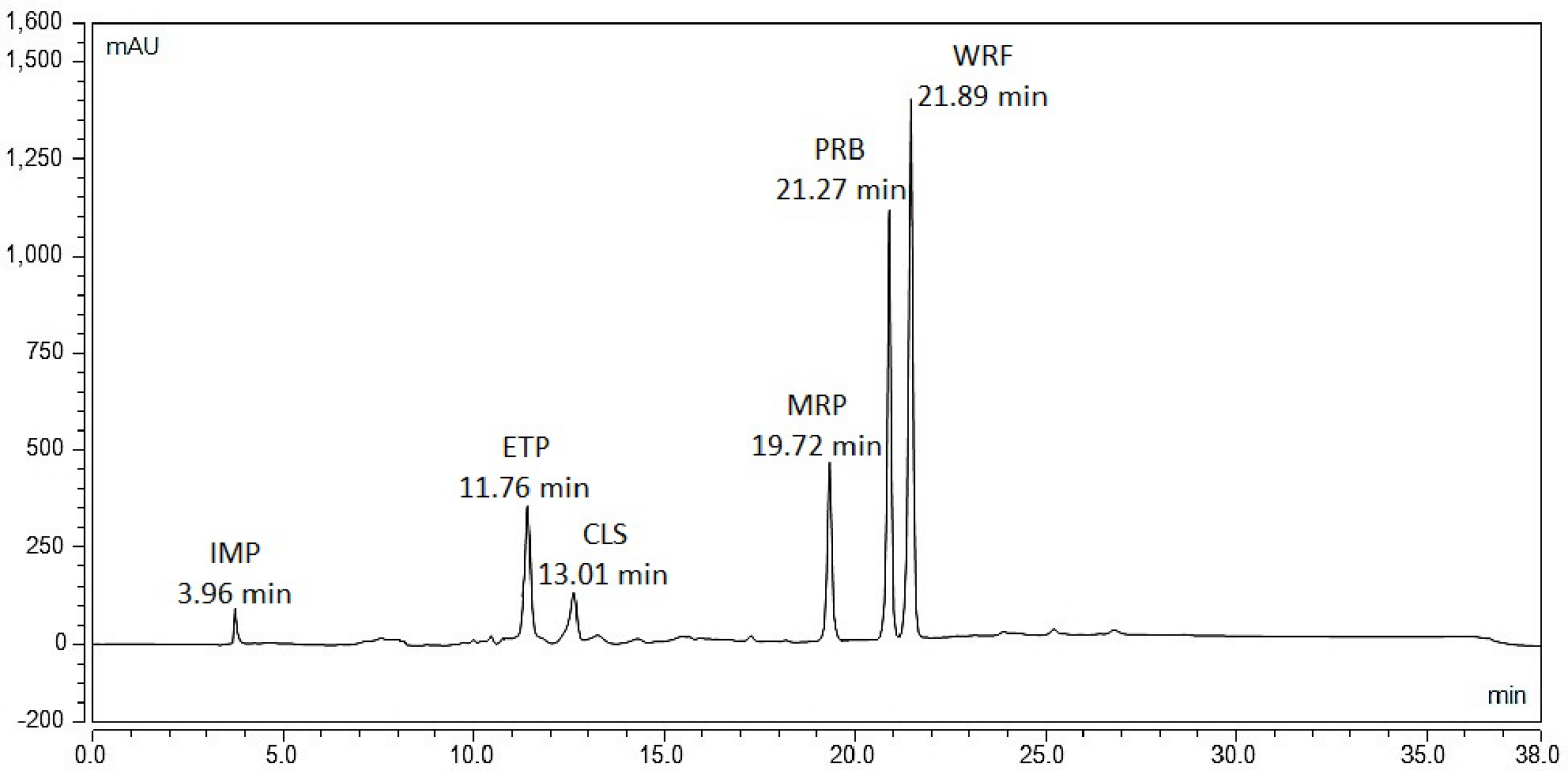
Chromatogram of the six drugs in plasma under the optimum conditions. Imipenem (100.0 µg/ml), Ertapenem (100.0 µg/ml), Cilastatin (100.0 µg/ml), Meropenem (100.0 µg/ml), Probenecid (100.0 µg/ml), Warfarin (100.0 µg/ml)

The pH was then investigated, beginning with pH 5.0 based on the information obtained from the predicted method, as well as pH 3.0 and 7.0 based on the pH studied in the design. Finally, the optimal pH was determined to be 3.0. By applying the optimum conditions, good resolution (R_s_) was achieved in which the lowest value is 1.80. As depicted in the chromatogram of the six drugs in plasma (Fig. 4), the retention times were as follows: IMP (3.38 min), ETP (11.67 min), CLS (12.88 min), MRP (19.59 min), PRB (21.14 min), and WRF (21.68 min).

### Method validation

#### System suitability

According to the results presented in Table 5, the studied parameters showed that the system is efficient.

**Table 5:** System suitability parameters for the developed RP-HPLC-PDA method

Parameters	Imipenem	Ertapenem		Cilastatin		Meropenem		Probenecid		Warfarin	Reference value
Retention time (t_R_) (min)	3.83	11.67		12.88		19.59		21.13		21.67	––
Capacity factor (K’)	2.83	10.67		11.88		18.95		20.13		20.67	> 2
Tailing factor (T_f_)	1.0	0.77		0.75		1.0		1.0		1.0	Less than 2
Asymmetry Factor (A_f_)	1.0	1.0		1.0		1.0		1.0		1.0	0.9–1.5
Resolution (Rs)	13.06		1.86		12.20		3.42		1.80		≥ 1.5
Column efficiency (N)	2704										> 2000

### Linearity and lower limit of quantification (LLOQ)

**Fig. 5:**
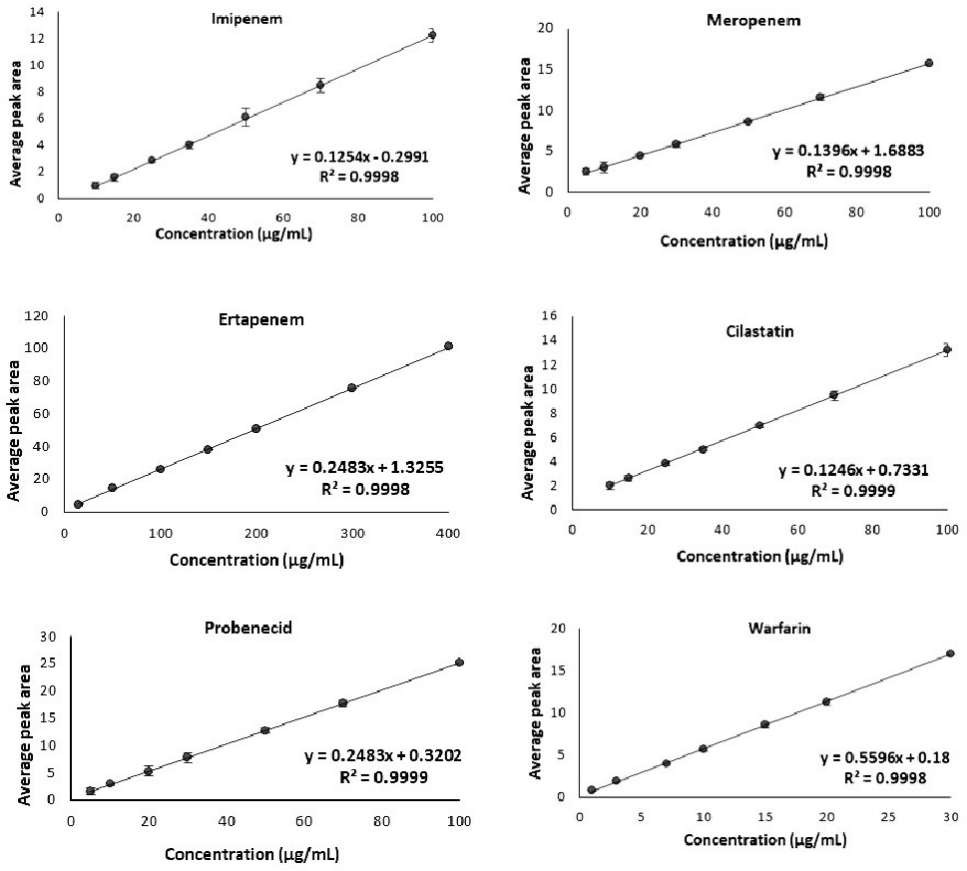
Calibration curves of the six drugs under investigation

Seven calibration points were selected for each of the six drugs to construct the calibration curve (Table 3). The drugs’ different calibration curve ranges were selected according to their Cmax. The method was linear in range of 10.0-100.0 µg/mL for IMP and CLS, 5.0-100.0 µg/mL for PRO and MRP, 15.0-400.0 µg/mL for ETP, and 1.0–30.0 µg/mL for WRF (Fig. 5). The resulted regression coefficients (R^2^) were near 1 which indicates good linearity for the six drugs. The generated regression equations and LLOQ concentrations are listed in Table 6.

**Table 6:** Linearity and LLOQ of the six drugs in spiked human plasma

Drug	Linearity (µg/mL)	Regression equation	R^2*^	LLOQ (µg/mL)
IMP	10.0–100.0	y = 0.1254x − 0.2991	0.9998	10.0
ETP	15.0 – 400.0	y = 0.2483x + 1.3255	0.9998	15.0
MRP	5.0–100.0	y = 0.1396x + 1.6883	0.9998	5.0
CLS	10.0–100.0	y = 0.1246x + 0.7331	0.9999	10.0
PRB	5.0–100.0	y = 0.2483x + 0.3202	0.9999	5.0
WRF	1.0–30.0	y = 0.5596x − 0.18	0.9998	1.0

^*^R^2^ is the regression coefficient

#### Selectivity

**Fig. 6:**
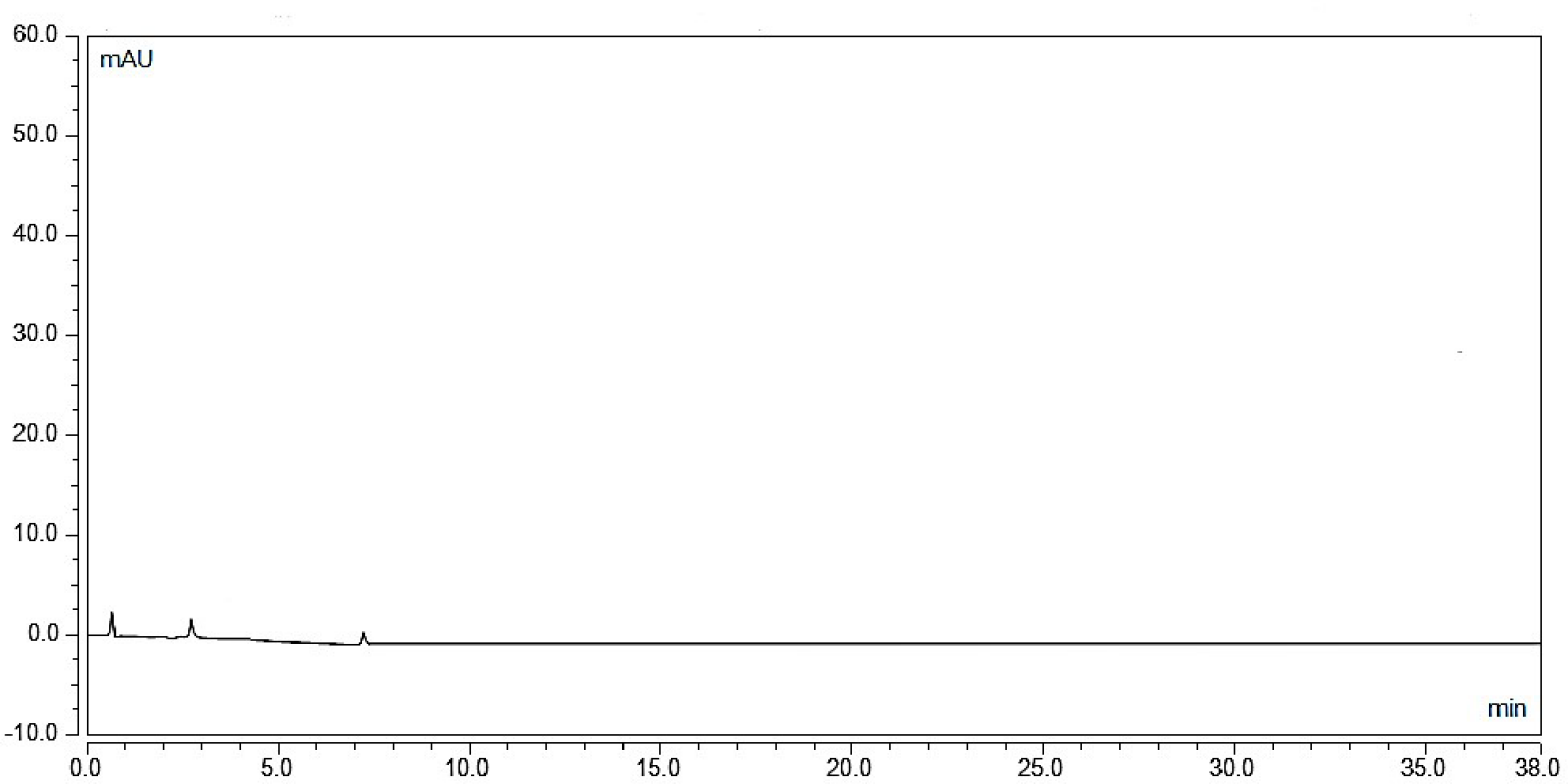
Chromatogram of blank plasma

As presented in Fig. 6, the method was proven to be selective as there was no interference from endogenous substances at the retention time of the drugs.

### Accuracy, precision and extraction recovery

The results in Table 7 shows that the method is accurate and precise with low % RSD and high % recovery. Mean within run accuracy (%R) and precision (%RSD) ranges were 98.56 − 100.56% and 0.19–1.88, respectively. Mean between run accuracy (%R) and precision (%RSD) ranges were 97.26 − 99.52% and 0.22–1.62, respectively.

**Table 7:** Accuracy and precision (within and between-run) of the six drugs in spiked human plasma

Drugs	Concentrations in plasma (µg/mL)		Within run (*n* = 3)		Between run (*n* = 12)	
			Mean accuracy (%R*)	Precision (%RSD**)	Mean accuracy (%R)	Precision (%RSD)
IMP	LLOQ	10.0	99.73	1.22	98.56	1.55
	QCL	30.0	99.03	0.68	98.45	1.08
	QCM	40.0	100.37	0.21	99.52	1.19
	QCH	75.0	99.94	0.77	99.24	0.66
ETP	LLOQ	15.0	99.09	0.69	98.75	0.33
	QCL	50.0	100.83	0.44	99.43	1.17
	QCM	140.0	99.79	0.28	98.64	0.99
	QCH	280.0	99.92	0.64	99.08	0.92
CLS	LLOQ	10.0	100.52	0.33	99.05	0.96
	QCL	30.0	99.35	0.86	98.63	0.81
	QCM	40.0	99.45	0.50	98.95	1.08
	QCH	75.0	100.28	0.55	99.15	0.93
MRP	LLOQ	5.0	99.19	1.88	98.37	0.22
	QCL	15.0	99.60	0.70	97.91	0.99
	QCM	35.0	99.69	0.68	99.17	0.44
	QCH	75.0	100.50	0.46	98.84	1.25
PRB	LLOQ	5.0	99.32	0.81	98.32	0.76
	QCL	15.0	99.72	0.40	98.27	1.37
	QCM	35.0	99.90	0.65	98.17	1.62
	QCH	75.0	100.04	0.60	98.35	1.50
WRF	LLOQ	1.0	98.56	0.19	97.72	0.63
	QCL	6.0	99.61	0.97	98.15	1.23
	QCM	12.0	98.14	0.31	97.26	0.83
	QCH	25.0	99.72	0.78	99.18	0.66

^*^%Recovery

^**^% relative standard deviation

Extraction recovery (%R) was evaluated for each of the six drugs, where the results ranged from 97.10 to 99.78% as shown in Table 8.

**Table 8:** Extraction recoveries of the six drugs in spiked human plasma

Drug	concentrations in plasma (µg/mL)		Extraction recovery (%)± %RSD
IMP	QCL	30.0	99.78 ± 1.60
	QCM	40.0	99.99 ± 0.83
	QCH	75.0	97.93 ± 1.68
ETP	QCL	50.0	97.22 ± 0.93
	QCM	140.0	97.07 ± 0.08
	QCH	280.0	98.81 ± 0.48
CLS	QCL	30.0	98.36 ± 1.18
	QCM	40.0	97.05 ± 1.41
	QCH	75.0	97.68 ± 0.34
MRP	QCL	15.0	98.74 ± 0.60
	QCM	35.0	97.21 ± 1.00
	QCH	75.0	98.09 ± 0.15
PRB	QCL	15.0	97.26 ± 1.32
	QCM	35.0	97.73 ± 1.02
	QCH	75.0	99.73 ± 1.02
WRF	QCL	6.0	98.62 ± 0.78
	QCM	12.0	99.73 ± 0.93
	QCH	25.0	98.38 ± 0.22

#### Stability

According to the results presented in Table 9, the method showed good stability results with low %RSD.

**Table 9:** Stability of the six drugs in spiked human plasma

Drug	concentrations in plasma (µg/mL)		Long term % dev ±%RSD	Benchtop % dev ±%RSD	Auto-sampler % dev ±%RSD	Freeze and thaw % dev ± %RSD
IMP	QCL	30.0	-7.36 ± 1.36	-2.35 ± 1.40	-1.13 ± 0.81	-3.91 ± 0.22
	QCM	40.0	-7.18 ± 1.51	-1.72 ± 1.40	-1.28 ± 1.17	-4.24 ± 1.48
	QCH	75.0	-6.88 ± 0.39	-2.79 ± 0.69	-1.56 ± 0.66	-4.55 ± 1.02
ETP	QCL	50.0	-7.34 ± 0.60	-2.73 ± 0.66	-1.87 ± 1.65	-4.88 ± 0.65
	QCM	140.0	-6.41 ± 0.63	-2.59 ± 0.80	-1.08 ± 0.16	-3.14 ± 0.10
	QCH	280.0	-7.86 ± 0.07	-1.94 ± 0.11	-1.26 ± 0.33	-3.18 ± 0.15
CLS	QCL	30.0	-6.76 ± 0.46	-1.80 ± 1.26	-1.27 ± 1.17	-4.28 ± 0.16
	QCM	40.0	-5.64 ± 0.66	-1.80 ± 0.48	-1.21 ± 1.06	-3.39 ± 0.46
	QCH	75.0	-6.21 ± 0.33	-2.96 ± 1.15	-1.91 ± 0.71	-4.20 ± 1.17
MRP	QCL	15.0	-5.85 ± 0.71	-2.57 ± 0.25	-1.68 ± 0.41	-3.13 ± 1.06
	QCM	35.0	-6.64 ± 1.19	-1.65 ± 1.04	-0.17 ± 0.11	-4.39 ± 0.55
	QCH	75.0	-5.36 ± 1.24	-3.60 ± 0.30	-2.94 ± 0.62	-4.39 ± 0.95
PRB	QCL	15.0	-6.98 ± 0.51	-2.49 ± 1.13	-1.62 ± 0.19	-4.16 ± 1.21
	QCM	35.0	-7.02 ± 1.06	-1.91 ± 1.76	-1.57 ± 0.72	-3.67 ± 0.80
	QCH	75.0	-4.79 ± 0.27	-2.15 ± 1.18	-0.57 ± 0.14	-3.12 ± 0.49
WRF	QCL	6.0	-6.89 ± 0.15	-2.12 ± 0.50	-1.26 ± 0.48	-3.94 ± 0.22
	QCM	12.0	-5.33 ± 0.14	-1.02 ± 0.76	-0.94 ± 0.89	-3.18 ± 0.55
	QCH	25.0	-4.11 ± 1.09	-1.83 ± 1.18	-0.90 ± 0.78	-2.78 ± 1.00

#### Application on human plasma

**Fig. 7:**
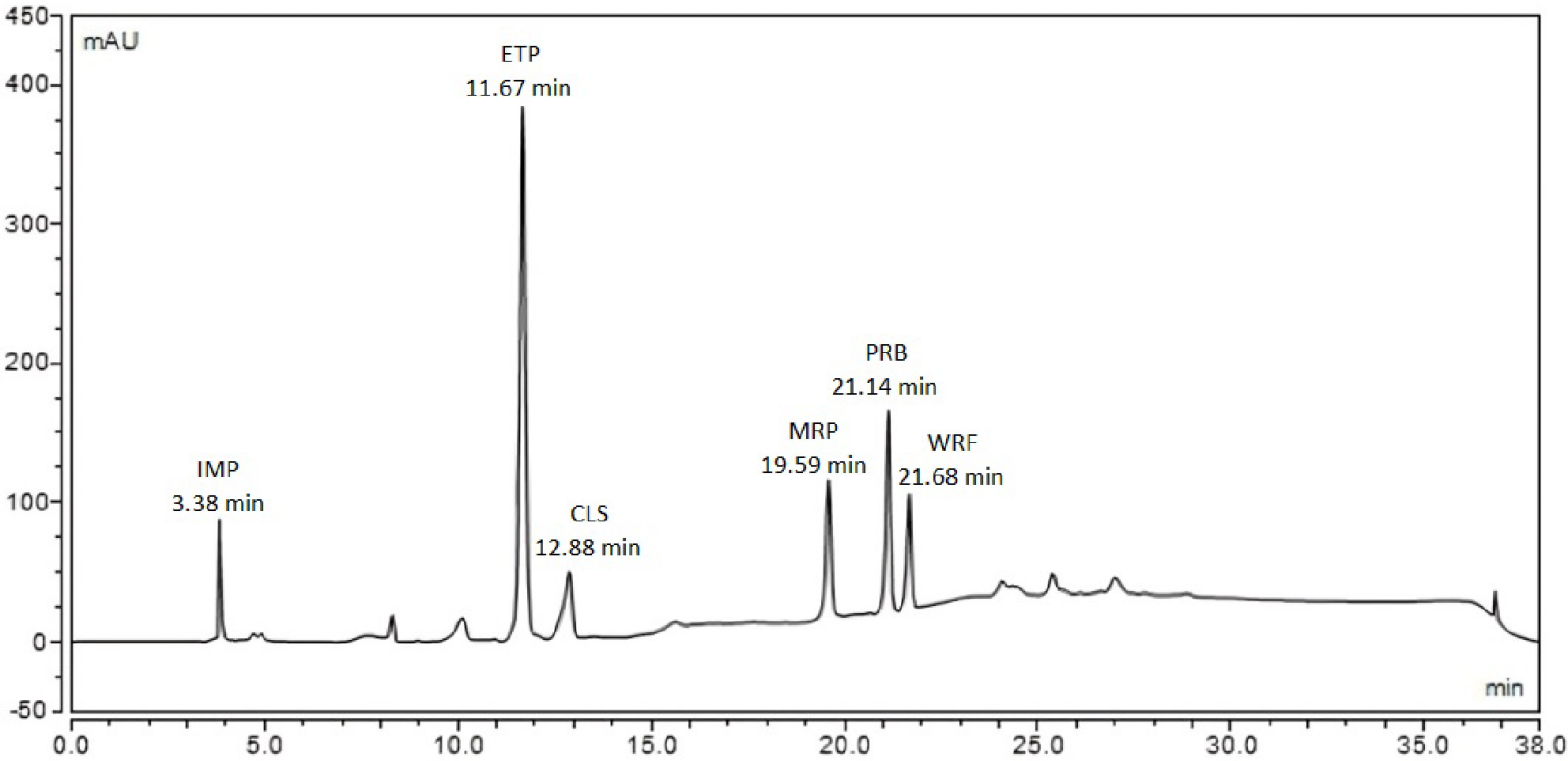
Chromatogram of the six drugs’ mixture in plasma at their Cmax concentrations under the optimum chromatographic conditions. Imipenem (35.0 µg/ml), Ertapenem (150.0 µg/ml), Cilastatin (35.0 µg/ml), Meropenem (30.0 µg/ml), Probenecid (30.0 µg/ml), Warfarin (10.0 µg/ml)

The method was successfully applied in plasma at the drugs’ Cmax levels which envisage its potential use in the selected drugs TDM assessments and clinical studies in the future by TDM laboratories. Figure 7 Shows the chromatogram of the drug mixture in plasma at their Cmax levels.

#### Greenness assessment

Despite the importance of many analytical procedures in controlling pollutants in the environment, they involve the usage of many solvents and reagents that can be hazardous to the environments. Green chemistry focuses on reducing the detrimental side effects of analytical methods and procedures, proper handling and detoxification of the analytical waste as well as minimizing the consumption of energy and hazardous reagents. In order to ensure the greenness of the analytical methods, different green assessment tools have been developed such as: National Environmental Methods Index, the Analytical Eco-scale, and a newly proposed tool called ‘the Green Analytical Procedure Index (GAPI). The latter was found to have more advantages over the other tools as it focuses on assessing the method for its waste production, energy consumption that the other tools ignores, and its health and environmental hazard impact [47, 48].

### Green analytical procedure index (GAPI)

To evaluate the greenness of the whole analytical method, the GAPI tool covers and studies all the analytical method stages from sample collection and preparation to the final analytical determinations and quantifications of the samples. A pictogram composed of five pentagrams was constructed to describe the greenness of the fifteen stages of the analytical method. The fifteen studied parameters included: sample collection, preservation, transportation, storage, type of method, scale of extraction, solvents/reagents used, additional treatments, the amount of reagents and solvents used, health hazard and safety hazard, energy consumption, occupational hazard, waste amount and waste treatment.

**Fig. 8:**
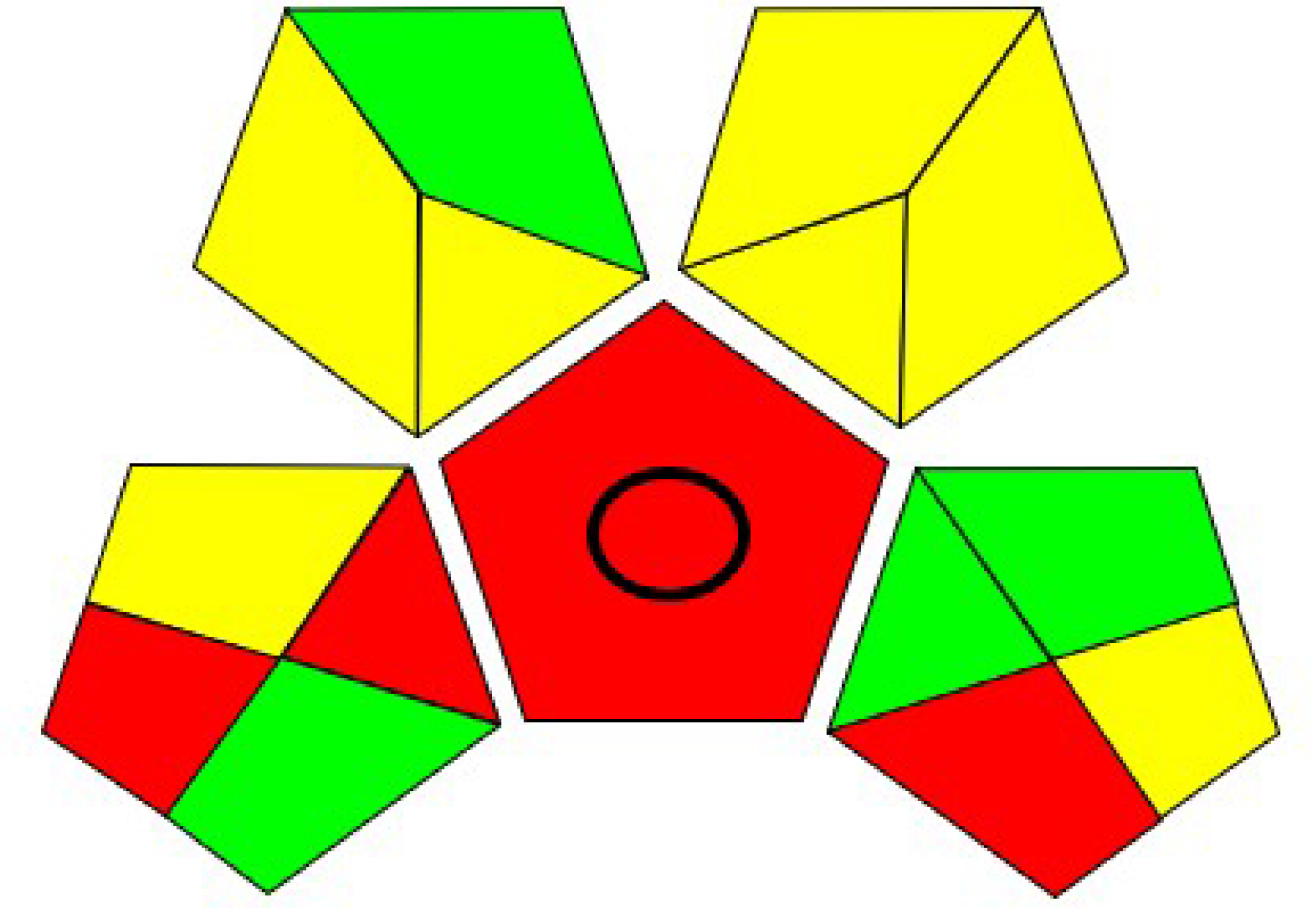
GAPI assessment for the proposed method

Every field in the pictogram was given a color code: green (= low), yellow (= medium), and red (= high) according to the environmental impact. The generated pictogram (Fig. 8) shows that sample collection which was given a red color code since it was an offline collection. No preservation was required, so it was given the green code. Transport was required, so it was given a red code. A yellow color code was given for the storage conditions field since sample preparation required normal storage conditions. Plasma extraction procedures were carried out in the method, so it was given a red color code. Moreover, since the scale of extraction was in microscale, the corresponding field was given a yellow color code. Yellow color was given to the solvents and reagents field due to the usage of green solvents such as MeOH. An additional sample treatment was required, so it was given a green color code. The amount of solvents used nearly ranged from 10 to 100 mL, so it was given a yellow color code. Since MeOH is considered moderately toxic and flammable with an NFPA score = 3, the corresponding field was given a yellow color. HPLC energy consumption was ≤ 1 kWh per sample, so it was given a green color. Occupational hazard was given a green color code. Low waste was produced (1–10 mL), so it was given yellow color. Finally, there was no waste treatment, so it was given red color code. Despite GAPI being a powerful tool in assessing the greenness of the method, there are no other reported methods for determining the six drugs to be compared with the proposed method to ensure the method’s greenness [48]. The Analytical Eco scale was implemented for collecting further data on the greenness of the method.

### The Analytical Eco-scale

It is considered a good semi-quantitative tool for assessing the greenness of the analytical methods, and ruling out the drawbacks of the analytical procedure. It mainly depends on giving penalty points to different parameters, and calculating them out of 100. A method with Eco-scale score greater than or equal 75 is considered an excellent green method. If the resulted Eco-scale score is more than 50, then it is an acceptable green method. And, if it is less than 50, then it is considered unacceptable and inadequate.

Four main parameters and factors are studied and given penalty points accordingly and they are: the amount of reagents, the amount of hazards, energy consumption, and waste production [47]. Table 10 shows that the calculated penalty points were more than 75 which signifies excellent green analysis of the developed method.

**Table 10:** The Analytical eco-scale penalty points of the developed RP-HPLC-PDA method

Parameters	Amount		Penalty points
Reagents			
*Methanol*	More than 100 mL		18
*Phosphate buffer*	Less than 10 mL		2
Energy consumption	Less than or equal to 0.1kWh per sample		0
Occupational Hazard	None		0
Waste	1–10 mL		3
Total penalty points			23
Eco-scale out of 100			77
Greenness of the method		Excellent green analysis	

## Conclusion

In this research, a green and simple method was developed for the determination of six drugs which are three carbapenems, imipenem and its DHP-I enzyme inhibitor: cilastatin, meropenem and ertapenem as well as two drugs that are contraindicated with them; probenecid and warfarin. The method was proven to be green by the help of two greenness assessment tools: GAPI and the analytical-eco scale. Bio-validation studies were carried out as per FDA guidelines, and the developed method was found to be specific, precise and accurate. Moreover, it was successfully applied on spiked human plasma at the drugs’ Cmax levels. The results show the feasibility of the method to be implemented for easy and direct TDM studies of these drugs when co-administered together in the future.

## Data Availability

Data and materials supporting this research are availible in the article.

## Abbreviations

TDM: Therapeutic drug monitoring
DDIs: Drug-drug interactions
IMP: Imipenem
MRP: Meropenem
ETP: Ertapenem
DHP-I: Renal dehydropeptidase-I
CLS: Cilastatin
PRB: Probenecid
WRF: Warfarin
HPLC: High performance liquid chromatography
UHPLC: Ultra high performance liquid chromatography
LC-MS/MS: Liquid chromatography- mass spectrometry
PDA: Photodiode array
Cmax: Maximum plasma concentration
GAPI: Green analytical procedure index
AES: Analytical Eco-scale
FDA: Food and Drug Administration
ACN: Acetonitrile
MeOH: Methanol
